# Sensitivity of Dried Blood Spot Testing for Detection of Congenital Cytomegalovirus Infection

**DOI:** 10.1001/jamapediatrics.2020.5441

**Published:** 2021-02-01

**Authors:** Sheila C. Dollard, Maggie Dreon, Nelmary Hernandez-Alvarado, Minal M. Amin, Phili Wong, Tatiana M. Lanzieri, Erin A. Osterholm, Abbey Sidebottom, Sondra Rosendahl, Mark T. McCann, Mark R. Schleiss

**Affiliations:** 1 US Centers for Disease Control and Prevention, Atlanta, Georgia; 2 Public Health Laboratory, Newborn Screening, Minnesota Department of Health, Saint Paul; 3 Division of Pediatric Infectious Diseases and Immunology, University of Minnesota Medical School, Minneapolis; 4 Division of Neonatology, University of Minnesota Medical School, Minneapolis; 5 Allina Health, Care Delivery Research, Minneapolis, Minnesota

## Abstract

**Importance:**

The sensitivity of dried blood spots (DBS) to identify newborns with congenital cytomegalovirus (cCMV) infection has not been evaluated in screening studies using the current, higher-sensitivity methods for DBS processing.

**Objective:**

To assess the sensitivity of DBS polymerase chain reaction (PCR) for newborn screening for cCMV infection using saliva as the reference standard for screening, followed by collection of a urine sample for confirmation of congenital infection.

**Design, Setting, and Participants:**

This population-based cohort study took place at 5 newborn nurseries and 3 neonatal intensive care units in the Minneapolis/Saint Paul area in Minnesota from April 2016 to June 2019. Newborns enrolled with parental consent were screened for cCMV using DBS obtained for routine newborn screening and saliva collected 1 to 2 days after birth. Dried blood spots were tested for CMV DNA by PCR at both the University of Minnesota (UMN) and the US Centers for Disease Control and Prevention (CDC). Saliva swabs were tested by CMV DNA PCR at the UMN laboratory only. Newborns who screened positive by saliva or DBS had a diagnostic urine sample obtained by primary care professionals, tested by PCR within 3 weeks of birth. Analysis began July 2019.

**Exposures:**

Detection of CMV from a saliva swab using a PCR assay.

**Main Outcomes and Measures:**

Number of children with urine-confirmed cCMV and the proportion of them who were CMV positive through DBS screening.

**Results:**

Of 12 554 individuals enrolled through June 2019 (of 25 000 projected enrollment), 56 newborns were confirmed to have cCMV (4.5 per 1000 [95% CI, 3.3-5.7]). Combined DBS results from either UMN or CDC had a sensitivity of 85.7% (48 of 56; 95% CI, 74.3%-92.6%), specificity of 100.0% (95% CI, 100.0%-100.0%), positive predictive value (PPV) of 98.0% (95% CI, 89.3%-99.6%), and negative predictive value (NPV) of 99.9% (95% CI, 99.9%-100.0%). Dried blood spot results from UMN had a sensitivity of 73.2% (95% CI, 60.4%-83.0%), specificity of 100.0% (100.0%-100.0%), PPV of 100.0% (95% CI, 91.4%-100.0%), and NPV of 99.9% (95% CI, 99.8%-99.9%). Dried blood spot results from CDC had a sensitivity of 76.8% (95% CI, 64.2%-85.9%), specificity of 100.0% (95% CI, 100.0%-100.0%), PPV of 97.7% (95% CI, 88.2%-99.6%), and NPV of 99.9% (95% CI, 99.8%-99.9%). Saliva swab results had a sensitivity of 92.9% (52 of 56; 95% CI, 83.0%-97.2%), specificity of 99.9% (95% CI, 99.9%-100.0%), PPV of 86.7% (95% CI, 75.8%-93.1%), and NPV of 100.0% (95% CI, 99.9%-100.0%).

**Conclusions and Relevance:**

This study demonstrates relatively high analytical sensitivity for DBS compared with previous studies that performed population-based screening. As more sensitive DNA extraction and PCR methods continue to emerge, DBS-based testing should remain under investigation as a potential low-cost, high-throughput option for cCMV screening.

## Key Points

**Question:**

What is the sensitivity of polymerase chain reaction testing for congenital cytomegalovirus deployed on dried blood spots obtained for universal newborn screening using current best methods?

**Findings:**

This cohort study of 12 554 newborns screened in a multisite study in Minnesota included 56 (4.5 per 1000) with confirmed congenital cytomegalovirus infection. The sensitivity of dried blood spots polymerase chain reaction testing was 85.7% with results of 2 laboratory results combined, which is substantially higher than reported in past studies.

**Meaning:**

The relatively high sensitivity of dried blood spots in the interim analysis of this study suggests their potential usefulness for universal cytomegalovirus screening as DNA extraction and polymerase chain reaction methodologies continue to improve.

## Teaser

This cohort study assesses the sensitivity of dried blood spots polymerase chain reaction for newborn screening for congenital cytomegalovirus infection using saliva as the reference standard for screening, followed by collection of a urine sample for confirmation of congenital infection

## Introduction

Congenital cytomegalovirus (cCMV), with an estimated birth prevalence of 4.5 per 1000 live births,^1^ is the most common congenital viral infection in the United States but remains underrecognized. Long-term outcomes associated with cCMV include sensorineural hearing loss, intellectual disability, cerebral palsy, seizure disorders, and learning delays that may develop in up to 20% of all infected children.^2,3^ Given the burden associated with cCMV and the proven benefits of treatment and early intervention for some affected infants,^4,5^ there has been growing interest in universal newborn screening.^6^

An optimal screening strategy for identifying newborns at risk for long-term cCMV-related sequelae remains uncertain. Congenital infection is currently diagnosed, in limited circumstances where cCMV infection is suspected, by performing polymerase chain reaction (PCR) for cytomegalovirus (CMV) DNA on infant urine, saliva, or (less often) blood, collected within 3 weeks of birth. Urine offers the highest sensitivity and specificity but is cumbersome to collect. Saliva provides high sensitivity although with lower specificity than urine.^7,8,9,10,11,12^ Because saliva is considerably more convenient to collect than urine, it is the preferred specimen for most cCMV screening studies with positive saliva result typically confirmed by urine testing.^7,8,9,13^ For universal cCMV screening, urine or saliva would both entail significant new expense and infrastructure for specimen collection and processing.

Dried blood spots (DBS) are already collected on virtually all newborns in the United States.^14^ However, CMV viral loads in blood are approximately 2 logs lower than viral loads in urine or saliva.^15^ The reported sensitivity of CMV testing on DBS compared with urine or saliva has varied widely depending on the methods used.^16,17^ The CMV and Hearing Multicenter Screening (CHIMES) study^18^ reported 34% DBS sensitivity for CMV compared with saliva in a subset of newborns enrolled from 2007 to 2008. Since then, diagnostic methods for CMV, including DNA extraction from DBS, have improved dramatically.^17^ From a public health standpoint, DBS need to be thoroughly evaluated with current best methods for cCMV screening before their utility is determined. In this study, we report interim findings of the analytical sensitivity of DBS for identifying newborns with cCMV infection using saliva PCR as the reference standard for screening, followed by collection of a urine sample for clinical confirmation.

## Methods

### Study Design

This prospective study was conducted at 5 Minneapolis/Saint Paul area newborn nurseries at the Fairview Health System (University of Minnesota [UMN] Masonic Children’s Hospital, Fairview Ridges Hospital, Fairview Southdale Hospital), and Allina Health System (Abbott Northwestern Hospital and United Hospital) and 3 neonatal intensive care units in the Fairview Health System. Parents of potential participants were approached by a study consenter from April 2016 to June 2019 (consenters were mainly present Monday through Friday) before hospital discharge, typically 24 to 48 hours after delivery. Newborns whose parent(s) provided written informed consent had a saliva specimen collected prior to discharge or within 2 weeks of birth (for infants in neonatal intensive care units). Saliva swabs were tested by the UMN laboratory within 1 week of collection. Dried blood spots used in this study were collected between 24 and 48 hours of age as part of routine newborn screening through the Minnesota Department of Health (MDH) Newborn Screening (NBS) Program. Punches from DBS of enrolled newborns were sent by MDH to UMN and the US Centers for Disease Control and Prevention (CDC) laboratories. Saliva and DBS test results from the UMN and CDC were reported directly to the MDH. The UMN and CDC laboratories were blinded to all clinical information on newborns and screening test results by the other laboratory until after their results were submitted to MDH. Staff from the MDH NBS Program contacted primary care professionals of newborns with a CMV-positive result by either saliva or DBS to discuss screening test results and to recommend follow-up urine testing within 3 weeks of birth and consultation with an infectious disease specialist. Cytomegalovirus PCR on urine specimens was performed at a Clinical Laboratory Improvement Amendments–certified diagnostic laboratory. The primary care professional notified the newborn’s family about the urine CMV test result and discussed follow-up options for newborns with confirmed cCMV. The study was approved by the institutional review boards at the UMN, Allina Health, MDH, and CDC.

### Specimen Collection

Polyester-tipped applicators (Puritan) with individual carrier tubes were used for collecting saliva samples from newborns. Swabs were placed in between the cheek and jaw and rotated for 5 seconds on each side then placed in tubes for transport to an area where swabs were air dried for 1 hour and stored at room temperature until transport to the UMN laboratory once per week for testing. Dried blood spots were collected as part of routine newborn screening and were provided to the study after written informed consent by the parents. Three 3-mm punches per well were punched into 96-well plates (Masterblock Deep Well Microplates; Greiner Bio-One) using the automated Panthera puncher (PerkinElmer). From each blood spot card, 2 sets of 3 DBS punches were prepared for transport to the UMN and CDC laboratories for testing.

### Laboratory Testing

Dried saliva swabs were processed for CMV PCR in the UMN laboratory. The first 4027 saliva swabs of the study were eluted with 300 μl of sterile distilled water as described in the CHIMES study.^13^ Because of early concerns with possible lower sensitivity with water elution, the method was changed to hydration of the dried swab in 300 μl of QuantaBio Extracta incubated at 95 °C for 30 minutes in a thermomixer with low agitation. Tubes were then chilled to 4 °C and centrifuged briefly. The eluate was used directly for PCR or stored at −80 °C until PCR testing. For PCR, 5 μl of eluate was used in a reaction volume of 25 μl. Primers, probes, and PCR conditions were carried out as previously described,^19^ using the LightCycler 96 PCR System (Roche). Internal controls for total DNA sample recovery were included as previously described.^19^ Polymerase chain reaction was run in duplicate and specimens were considered positive if 2 of 2 replicates were positive or if 3 of 4 replicates were positive for samples tested twice. For calculating copies per millileter of saliva, the volume of saliva eluted from the swab was estimated to be 75 μl based on previous studies.^20^ Polymerase chain reaction quantitation of CMV for saliva and DBS in both laboratories used the World Health Organization International CMV Standard.^21^

For DBS processed at the UMN, DNA was extracted from three 3-mm punches using the QIAcube HT extractor with the QIAamp 96 DNA kit (Qiagen) with slight modifications. Briefly, 240 μL of Buffer ATL (Qiagen) with 10% proteinase K solution was added to each DBS sample and digested overnight in a Thermomixer (Eppendorf) incubator at 56 °C at 400 revolutions per minute (rpm). The material was then transferred to a deep-well 96 well plate (S-block; Qiagen), and automated extraction was done with the QIAamp (Qiagen) 96 DNA version 1 protocol slightly modified to elute with 100 μL of water. Polymerase chain reaction testing of DBS DNA was performed in triplicate as previously described^19^ with 10 μl of eluate. A DBS was considered positive for CMV if at least 2 of 3 replicates were positive or at least 3 of 6 for specimens tested twice. Viral loads were expressed as international units per millimeter of blood. To calculate international units per millimeter of blood for DBS, blood volumes were extrapolated from the diameter of DBS punches based on established guidelines.^22^

Dried blood spot DNA extraction at the CDC laboratory was a quick manual process using Quanta Extracta DBS buffer (QuantaBio). Dried blood spots in 96-well plates were washed by adding 200 μl Quanta DBS buffer per well and centrifuging plates at 3500 rpm for 5 minutes. Buffer was removed and discarded and 60 μl fresh buffer per well was added and incubated at 95 **°**C on a thermomixer for 25 minutes without shaking. Plates were rapidly cooled on a prechilled block then centrifuged at 3500 rpm for 5 minutes. Eluate was used directly for PCR. Cytomegalovirus PCR was performed using PerfeCTa Fastmix II Low ROX master mix (QuantaBio) with TaqMan internal positive control to monitor for inhibition (ThermoFisher). The CMV Taqman probe MGBNFQ (ThermoFisher) and primers (Integrated DNA Technologies) targeted the viral immediate early region.^13^ The human gene ribonuclease P was amplified separately to monitor DNA extraction performance. Specimens were tested in triplicate using the Aria MX thermocycler (Agilent) and results interpreted as described above for the UMN method. The sensitivity of the assay was 5 or fewer copies CMV/PCR reaction.

### Sample Size Calculation

Our projected enrollment sample of 25 000 newborns was based on the group sample sizes required to evaluate DBS clinical sensitivity, ie, the ability of a test to identify cases of cCMV disease present at birth or that manifests by age 3 to 4 years. We hypothesized that DBS would identify most cases of cCMV disease; thus, the clinical sensitivity of DBS would approximate the sensitivity of saliva, despite the lower DBS analytical sensitivity. We assumed the analytical sensitivity of DBS compared with saliva screening was 60%, and the proportion with cCMV disease was 17.5% among newborns detected by saliva screening and 37.5% among those detected by DBS screening. Group sample sizes of 90 newborns with cCMV detected by saliva screening and 54 by DBS screening would achieve 80% power to detect a 20% difference between the group proportions of disease, with α of .05, using the 1-sided Mantel-Hanzel test. We assumed cCMV prevalence was 4.5 per 1000; thus, to achieve a sample size of 90 newborns with confirmed cCMV identified by saliva screening in the final study, considering withdrawals and loss of follow-up, we aimed to screen 25 000 newborns.

### Data Analysis

Because saliva was considered a reference standard for cCMV screening, confirmatory urine CMV testing was only collected from infants by the primary care professionals for those infants who had a CMV-positive screening result in either saliva or DBS. For assessing test performance, we only included CMV-positive newborns by either positive saliva or DBS testing with a follow-up urine testing and assumed those with negative saliva and DBS tests in both laboratory results were true negatives. We calculated sensitivity and specificity for saliva PCR and DBS PCR in combined and individual laboratory results. We also calculated the proportions of true-positive (predictive value positive), false-positive, true-negative (predictive value negative), and false-negative results. We estimated 95% CIs using the Wilson Score method. Statistical analyses were performed using Open Epi version 3. Analysis began July 2019.

## Results

### Study Population and Prevalence of cCMV

The consent rate for parents approached for the study was 70% (12 554 of 17 822). The demographic characteristics of 12 554 newborns included in this interim analysis are shown in Table 1; 9480 (75.5%) were born to non-Hispanic White mothers. Saliva swabs were collected within 3 days of life in 11 968 screened newborns (95.3%) and within 3 weeks of life for the remaining 586 (4.7%). Among screened newborns, 65 (0.52%) were CMV positive for saliva, DBS, or both. When CMV screen-positive results were reported to the primary care professional, 64 of 65 families (98%) agreed to follow-up urine testing. Fifty-six newborns were CMV positive for urine and confirmed to have cCMV for a prevalence of 4.5 (95% CI, 3.4-5.8) per 1000 live births.

**Table 1.** Characteristics of Newborns Screened for Congenital Cytomegalovirus Infection in Minnesota

Characteristic	No. (%)	
	Total screened from 2016-2019	Minnesota live births from 2016-2018^a^
Overall	12 554 (100)	205 688 (100)
Maternal age group, y		
≤24	1145 (9.1)	35 355 (17.9)
25-29	2786 (22.2)	62 193 (30.1)
30-34	5353 (42.6)	70 533 (34.6)
≥35	3234 (25.8)	37 607 (18.3)
Unknown	36 (0.3)	
Mother’s race		
White	9480 (75.5)	154 982 (75.3)
Black	1274 (10.1)	25 303 (12.3)
Asian	925 (7.4)	16 547 (8.0)
American Indian/Alaska Native	117 (0.9)	3499 (1.7)
Unknown/other^b^/mixed	758 (6.0)	5357 (2.6)
Parity^c^		
1	5316 (42.6)	73 556 (35.8)
2	3998 (32.0)	66 278 (32.2)
≥3	2273 (18.2)	65 565 (31.9)
Unknown	967 (7.7)	289 (0.1)

^a^ Data are from the US Department of Health and Human Services, US Centers for Disease Control and Prevention, Natality public use data.^23^

^b^ Other race comprised multiracial, marked as unknown, or left blank by the mother or parents.

^c^ Birth order for Minnesota live births.

### Test Performance

Of 56 newborns with confirmed cCMV, 44 (78.6%) tested positive by both saliva and DBS, 8 (14.3%) tested positive by saliva only, and 4 (7.1%) tested positive by DBS only. The sensitivity was 85.7% (48 of 56) for DBS testing with UMN and CDC results combined, 73.2% (41 of 56) by UMN DBS testing, and 76.8% (43 of 56) by CDC DBS testing (Table 2). Among the 49 DBS screen-positive newborns, 1 had CMV-negative urine and was determined to be false positive for a total of 2.0% (1 of 49) false-positive DBS results. This DBS was later sent to the CDC blinded and retested as CMV negative. Results according to specimen type are summarized in Table 2.

**Table 2.** Performance of DBS and Saliva Polymerase Chain Reaction Testing for Identifying Newborns with Congenital CMV Infection (N = 12 554)

Congenital CMV infection^a^	Saliva		DBS combined		DBS UMN		DBS CDC	
	Yes	No	Yes	No	Yes	No	Yes	No
Positive screen, No. (%)	52 (0.4)	8 (0.1)	48 (0.4)	1 (0)	41 (0.3)	0 (0)	43 (0.3)	1 (0)
Negative screen, No. (%)	4 (0)	12 490 (99.5)	8 (0.1)	12 497 (99.5)	15 (0.1)	12 498 (99.6)	13 (0.1)	12 497 (99.5)
**Parameter, % (95% CI)**	**Saliva**		**DBS combined**		**DBS UMN**		**DBS CDC**	
Sensitivity	92.9 (83.0-97.2)		85.7 (74.3-92.6)		73.2 (60.4-83.0)		76.8 (64.2-85.9)	
False negative	7.1 (2.8-17.0)		14.3 (7.4-25.7)		26.8 (17.0-39.6)		23.2 (14.1-35.8)	
Specificity	99.9 (99.9-100)		100.0 (100-100)		100.0 (100-100)		100.0 (100-100)	
PPV	86.7 (75.8-93.1)		98.0 (89.3-99.6)		100.0 (91.4-100)		97.7 (88.2-99.6)	
False positive	13.3 (6.9-24.2)		2.0 (0.4-10.7)		0.0 (0.0-8.6)		2.3 (0.4-11.8)	
NPV	100 (99.9-100)		99.9 (99.9-100)		99.9 (99.8-99.9)		99.9 (99.8-99.9)	

Abbreviations: CDC, US Centers for Disease Control and Prevention; CMV, cytomegalovirus; DBS, dried blood spots; NPV, negative predictive value; PPV, positive predictive value; UMN, University of Minnesota.

^a^ Yes indicates congenital CMV infection confirmed by urine polymerase chain reaction test in newborns with a positive screening result in either saliva swab or DBS by at least 1 laboratory. No indicates congenital CMV infection ruled out by urine polymerase chain reaction test in newborns with a positive screening result or newborns with negative screening results for saliva and DBS at both laboratories.

Among 60 saliva screen-positive newborns, 8 had CMV-negative urine and were determined to be false positives, for a total of 13.3% (8 of 60) false-positive saliva results. Four newborns had false-negative CMV saliva results but screened positive by DBS and were confirmed positive by urine; therefore, the saliva was 92.9% (52 of 56) sensitive (Table 2). This prompted additional testing of saliva specimens to identify possible technical explanations. Three of 4 false-negative saliva swabs were among the first 4027 specimens of the study that were eluted with water. The first specimen had an extremely high CMV viral load, presumably causing PCR inhibition, and after 10-fold dilution was CMV positive. The second specimen was CMV positive after the swab was transferred from water to quanta buffer. The third specimen had very few human cells indicating inadequate sample collection. The fourth specimen had no technical problem identified and remained CMV DNA negative in several replicate tests. To further troubleshoot false-negative saliva results, we compared levels of cellular DNA from swabs eluted with water to swabs eluted with Quanta buffer. Recovery of cellular DNA with water was 87% lower than with Quanta (mean [interquartile range], 2.3 × 10^3^ [4.05 × 10^2^ to 9.74 × 10^3^] cells per mL for water vs 9.2 × 10^5^ [8.65 × 10^5^ to 1.07 × 10^6^] cells per mL for Quanta [*P* < .001]).

### CMV Viral Load in Saliva and DBS

Viral loads for all screen-positive samples are shown in the Figure. The median (interquartile range) viral load for the 60 positive saliva samples was 1.34 × 10^6^ IU/mL (1.87 × 10^3^ to 3.09 × 10^7 ^IU/mL). The median (interquartile range) viral load for the 49 positive DBS samples was 6.6 × 10^3^ IU/mL (2.97 × 10^3^ to 1.60 × 10^4^ IU/mL). Values for DBS that were positive in both the CDC and UMN laboratories are the mean IU/mL value for that newborn.

**Figure.**
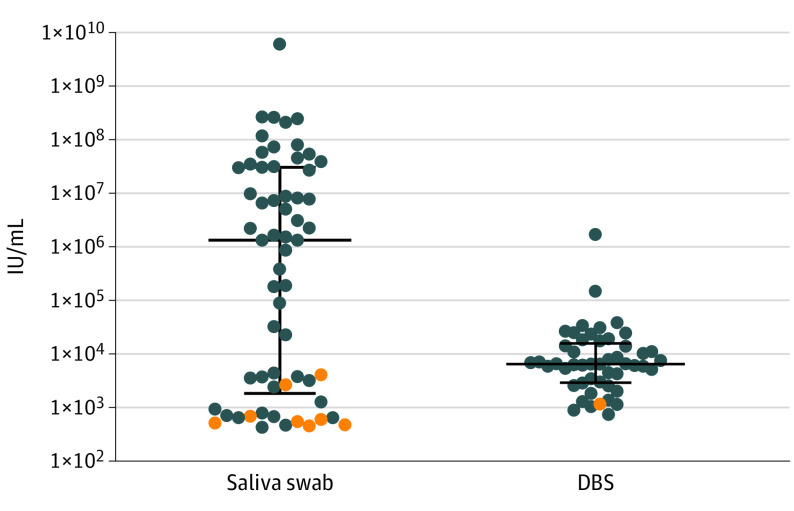
Distribution of Cytomegalovirus Viral Load for All Screen Positive Results for Saliva (n = 60) and for Dried Blood Spots (DBS) (n = 49) Blue circles show viral load values for urine-confirmed cases of congenital cytomegalovirus infection for saliva (n = 52) and DBS (N = 48). Orange circles show viral load values for false-positive results for saliva (n = 8) and DBS^1^ in newborns with cytomegalovirus-negative urine. Results are expressed as international units (IU) per millimeter of saliva or blood. The median viral load value was 1.34 × 10^6^ IU/mL (interquartile range, 1.87 × 10^3^ to 3.09 × 10^7^ IU/mL) for saliva and 6.6 × 10^3^ IU/mL (interquartile range, 2.97 × 10^3^ to 1.60 × 10^4^) for DBS. Horizontal lines show the median viral load, and the error bars represent interquartile range.

## Discussion

In this article, we describe interim findings of analytical sensitivity of DBS PCR for cCMV screening based on an unselected cohort of 12 554 newborns enrolled in a multisite study in the Twin Cities of Minneapolis and Saint Paul in Minnesota. The main finding of our study was the enhanced sensitivity of DBS PCR for identification of cCMV (86% for combined laboratory results 73% and 77% for individual results from UM and CDC, respectively) compared with previous studies.^18^ The main reasons for increased sensitivity are most likely because of improved methodologies. Specifically, the DNA extractor introduced by Qiagen in 2003 used in CHIMES^18^ was shown to yield considerably less DNA than other purification systems.^17^ The Quanta DBS elution buffer used by the CDC laboratory has high DNA yield, is fast and inexpensive,^23^ and is currently used by several state NBS programs (including Colorado, Michigan, Minnesota, Wisconsin, and Washington) for DNA-based testing.^24^

Our study assessed the prevalence of cCMV in a Midwestern US population, which was predominantly non-Hispanic White.^25^ The CHIMES Study^18^ showed that cCMV prevalence varies substantially by race and Hispanic origin: 9.5 per 1000 among Black newborns and 2.7 per 1000 among non-Hispanic White newborns. Despite a higher proportion of non-Hispanic White newborns in our study compared with the CHIMES population (75.5% vs 37.1%), our cCMV prevalence was similar at 4.5 per 1000.^13^ Our sample size is currently too small to provide reliable estimates for demographic subgroups. A larger sample size and more data will be available in 2 years, which will help clarify risk factors and clinical outcomes for cCMV in our cohort.

Our study offers useful observations on acceptability and feasibility of cCMV screening. Despite the newborn period being hectic and stressful for parents, 70% of those approached consented, consistent with surveys reporting generally positive parental attitudes toward cCMV screening.^26,27^ We noted that there were minimal barriers in communicating positive screen results to primary care professionals. Once notified of a positive result, clinicians and families were highly compliant with returning for follow-up urine testing. These observations support the potential acceptability and utility of incorporating cCMV screening into state health department NBS programs.

### Limitations

Although our study was designed to use saliva as the reference standard, saliva screening produced both false-positive and false-negative results, similar to results from other studies.^5,9^ The proportion of positive saliva results in our study that were false positive (13.3%) is similar to that reported for other studies that performed cCMV screening with saliva.^9,10^ False-positive saliva results are most likely related to colonization of the neonatal oropharynx with CMV DNA from colostrum.^28^ Among the 12 554 newborns screened in our study, 8 saliva results were false positive, corresponding to a false positive rate of 0.06%, which is comparable with the false positive rate of 0.03, 0.14% described in the CHIMES cohort.^29^ The recent US Food and Drug Administration approval of a point-of-care neonatal saliva CMV test (Meridan Bioscience), underscores the importance of further clarifying the role of false-positive saliva CMV test results and underscores the requirement for urine confirmation for diagnosis of cCMV.^9,30^

## Conclusions

In conclusion, our interim findings provide a basis for further development of DBS PCR as a screening modality for cCMV. Although the 75% average sensitivity of DBS for the 2 assays in our study remains suboptimal for screening, further improvements in methodologies for DNA extraction and PCR should allow for better sensitivity with the individual tests. The clinical sensitivity of DBS, ie, the ability to identify newborns with or at high risk for cCMV, remains to be determined and may be higher than the analytical sensitivity. Our study will follow-up infants with confirmed cCMV for up to 4 years of age to assess for all clinical outcomes. Diagnostic methods are always improving, and therefore, our results show the potential of DBS to provide low-cost CMV screening with smooth integration of sample collection, laboratory testing, and follow-up.^14^
